# Differential temporal salience of earning and saving

**DOI:** 10.1038/s41467-018-05201-9

**Published:** 2018-07-20

**Authors:** Kesong Hu, Eve De Rosa, Adam K. Anderson

**Affiliations:** 1 Department of Human Development, Cornell University, Ithaca, 14853-4401 NY USA; 2 Human Neuroscience Institute, Cornell University, Ithaca, 14853-4401 NY USA

## Abstract

People are often characterized as poor savers. Here we examined whether cues associated with earning and saving have differential salience for attention and action. We first modeled earning and saving after positive and negative variants of monetary reinforcement, i.e., gains versus avoiding loss. Despite their equivalent absolute magnitude in a monetary incentive task, colors predicting saving were judged to appear after those that predicted earning in a temporal-order judgment task. This saving posteriority effect also occurred when savings were framed as earnings that come slightly later. Colors predicting savings, whether they acquired either negative or positive value, persisted in their posteriority. An attentional asymmetry away from money-saved relative to money-earned, potentially contributes to decreased everyday salience and future wealth.

## Abstract (web summary)

Economists have observed that many people seem unwilling to save for the future. Here, the authors show that earning and saving are subject to a basic asymmetry in attentional choice, such that cues that are associated with saving are perceived as occurring later than cues associated with earning.

## Introduction

In the parable of the ant and the grasshopper, the ant’s assiduous collection of food, saving for the winter, contrasts with the grasshopper’s pursuits of immediate gratification. Cast more as grasshopper than ant, humans are often characterized as poor savers. This reputation may be well earned, in particular in America. According to a 2016 analysis of the Federal Reserve’s 2013 survey of consumer finances, the median American working-age couple has saved only $5000 for retirement with 43% of working-age families estimated to have no retirement savings at all^1^. On a long downward trend, the personal savings rate (expressed as a percentage of disposable personal income, DPI), dropped to <3% of DPI at the close of 2017^2^. Contrasting with a near 96% employment rate, we have ant-like work ethic, yet earnings are rarely converted into savings.

Here we assess, in the context of monetary reinforcement, whether earning and saving reflect an asymmetry in value-derived attention^3–5^, asking whether the attentional scales are tipped in one’s favor. The way we attend has important interactions with value, perception, decision making, and ultimately behavior^3^. To assess their comparative behavioral and attentional salience, we considered two potential conceptual models for the distinction between earning and saving. First, we modeled earning and saving after positive and negative reinforcement, i.e., gains versus avoiding losses. Second, we considered earning and saving as variants of positive reinforcement in which gains accumulate at the same rate, but differ according to a conceptual framing of manifesting immediately or a short time later.

Inspired by language, one meaning of “to save” is to avoid loss. Saving may represent an aversion to losing one’s earnings. The assessment of gains and losses is central to our most basic physiological needs and drives^6^. There are evident asymmetries in the weight we place on gains and losses, with potential losses having an incommensurate influence when people evaluate identical outcomes^7^. In field experiments, monetary incentives framed as losses (“avoid losing A by doing B”) increases factory workers’ productivity relative to those as gains (“gain A by doing B”)^8^. Such loss aversion has received empirical support from a variety of studies^9–12^, and when directly experienced, losses outweigh gains^13^. Loss aversion is related to biases such as the endowment effect^14^ and the status quo bias^15^, suggesting that individuals should place greater value on savings they have already earned. But quite to the contrary, poor saving behavior^16^ suggests loss aversion is likely not at work in limited savings. One must be motivated to accrue savings before being concerned about losing them.

Loss aversion and related biases are thought to reflect the asymmetric weighting of punishment and reward^17^. Losses are punishing, resulting in an exaggerated avoidance response, biasing both decisions and the amount of attention devoted to them^17,18^. While the act of avoiding loss is the removal of punishment, and thus is reinforcing^19^. Motivation to earn versus save may, more directly, be a comparison of positive and negative variants of reinforcement^20^, comparing earnings with the avoidance of losing one’s earnings. Positive and negative reinforcement refer to increasing the likelihood of a behavior with the addition or subtraction of an outcome, not their positive or negative utility for an individual. Positive and negative reinforcement have been shown to similarly recruit the reward system, suggesting that both have positive utility^21^. Nevertheless, they may have asymmetric motivational power^12^. Psychologically, and in our daily experience, individuals believe they are paid for their performance rather than arranging conditions to avoid moneyless periods of time^22^. Savings, in this context, should motivate individuals to avoid being without money. Earning and saving should then align with different concerns. Moreover, individuals can differently experience pleasure or utility according to their promotion versus prevention orientation^23^, through either promoting desired versus preventing undesired outcomes.

On the other hand, the meaning of “to save” could be understood in terms of expected utility in the future, hence currently inaccessible. In line with this, efforts to avoid moneyless periods of time highlight the importance of temporal perspectives on one’s earnings. Maintaining an orientation toward saving may result in temporal discounting of today’s earnings in the future^24^. Discounting of future value is captured by individuals who prefer $5 now compared to $10 three months from now^25,26^. Participants often make choices of smaller but immediate rewards relative to rewards that are larger but delayed. Such temporal or delay discounting is also considered a marker of impulsive behavior, assessing the degree to which the subjective value of an offering decreases as a function of delay in its delivery^27^. While the rate of discounting depends on the individual, it is a fundamental to the representation of value, observed in human and nonhuman animals^28^. Temporal distance of saving for the future may also modulate value representations such that they are more abstract^29^. This may cognitively distance individuals from the reality of the undesirable outcomes of not saving, i.e., extended moneyless periods of time. Saving in these contexts reflects an orientation toward the future, as well as the limits of imagination on behavior^30^. Accordingly, while earning may reflect the here and now, savings may reflect earnings as a discounted and abstract future.

Whether earning and saving reflect varieties of reinforcement or differentially reflect temporal discounting, they involve making a choice between options^31^. Our nervous system is confronted, at each moment, by choices in terms of where to invest or allocate its resources in the currency of attention^3,32^. By paying attention, an individual is able to impact the salience^33^ and value^34^ of sensory events. While unpleasant events typically evoke relatively stronger changes in affect and attention in both perceptual and decision studies^9,12,17,18,35,36^, gains also play a similar role^3,4,37–39^. Importantly, value not only alters attention, but attention is also central to value, with attention-boosting^34^ and inattention-reducing value^40^. Attention can both follow and influence preference^41^, predicting consumer choice^42^. Thus, the choice to what we attend is central to value and behavior^17,18,43^. While multiple studies have characterized value-derived attention^3–5^, much less is known about how different variants of reinforcement and temporal framing regulate attention. Here we examined how earning and saving, according to different models, regulate the paying of attention. If earning and savings represent differential concerns to the individual, then this should be reflected in attentional choice, having an asymmetric regulatory influence on salience and awareness.

Mirroring how value is scaled relative to time^44^, time is also scaled relative to attention. Attention shapes not only what is perceived but also when^45^. Attention can warp the judgment of temporal order, with attended events appearing to occur before non-attended events, called “prior entry”^46,47^. Similarly, individuals attend to more immediate events and outcomes than those in the more distant future^29^, and this asymmetry in attention may modulate temporal discounting^48^. We took advantage of how attention can influence judgments of temporal order to examine how individuals perceive events predicting earning and saving. Just as how individuals may put off saving due to decreased salience and relative inattention, monetarily reinforced colors associated with savings may be less attentionally salient and appear to come later. As a model for earnings and savings, we first examined the power of positive (gains) and negative (avoiding losses) monetary reinforcement of color patches and the relationship between action and attentional salience (experiments 1a–c). In a further study (experiment 2), through distinct temporal framings of positive reinforcement, we modeled earning and saving after gains that come immediately versus gains to come later (i.e., saving for future).

**Fig. 1:**
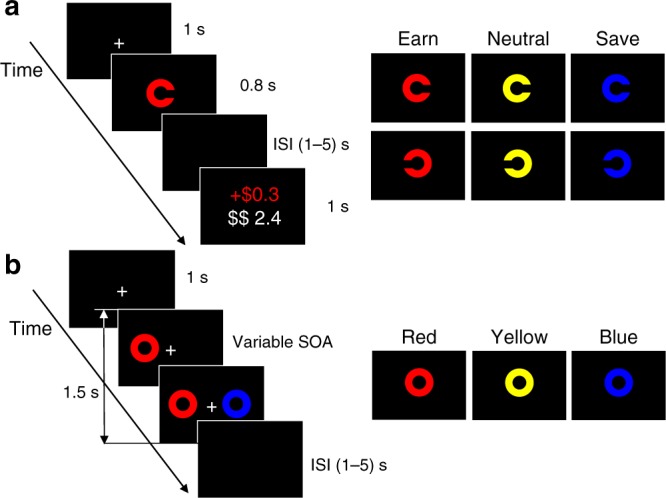
Illustration of the display sequences and target stimuli examples. **a** Monetary reinforcement task. Exp. 1a involved a color discrimination (red, blue, and yellow), while Exp. 1b and 1c involved a gap side (left and right) discrimination. After response, participants were informed about gain or loss, together with the total cash bonus accrued (in white). **b** Temporal-order judgment task. Following fixation, colored circles were presented either on the left or on the right side of the fixation followed by a second different color circle, which appeared on the opposite side after a variable SOA (8, 18, 38, 68, and 98 ms). Participants were required to indicate which color (Exp 1a) or which side (Exp 1b and 1c) appeared first

Figure 1 illustrates the core tasks and the example colored circle used as stimuli. Participants started with value reinforcement trials, where equiluminant colors (red, blue, or yellow) were 100% reinforced, or received no reinforcement, for fast and accurate color discriminations. One color was associated with “earning,” gaining 30 cents, and another associated with “saving,” avoiding loss of 30 cents. The task was sufficiently easy to enable reinforcement on the majority trials, whereby earning would increase one’s balance and saving would preserve those earnings. Participants received their performance-based earnings at the end of the experiment. Color-reinforcement associations were counterbalanced across participants. The temporal-order judgment (TOJ) task required participants to judge which of the side-by-side colored stimuli appeared first, when presented in varying temporal proximity (8–98 ms). TOJ trials were pseudo-randomly intermixed with value reinforcement trials to ensure that any acquired salience for colors was maintained throughout. Similar to indifference points in temporal discounting to establish value^25,26^, we estimated the participant’s point of subjective simultaneity (PSS), which indicates the estimated time interval to perceive the two stimuli as arriving simultaneously, i.e., 50%^47,49–51^.

Despite their equivalent absolute magnitude in a monetary incentive task, we find that saving results in less behavioral salience and decreased payout. In a temporal-order task, saving-associated color cues are also judged to appear after those that predict earning, consistent with the decreased attentional salience of saving. This saving temporal posteriority effect generalizes to when saving is framed as earnings that come slightly later. Across studies, saving-associated cues persisted in their relative inattention whether the cues acquire negative or positive valence. Thus, saving posteriority is not simply explained by acquired affective value. We conclude that decreased attentional salience related to money-saved relative to money-earned is a fundamental information-processing bias. That saving has less moment-to-moment attention attracting potential may contribute to reduced saving behavior. Attentional interventions to enhance the everyday salience of saving may be gainfully employed to improve saving behavior.

## Results

### Experiment 1a

**Fig. 2:**
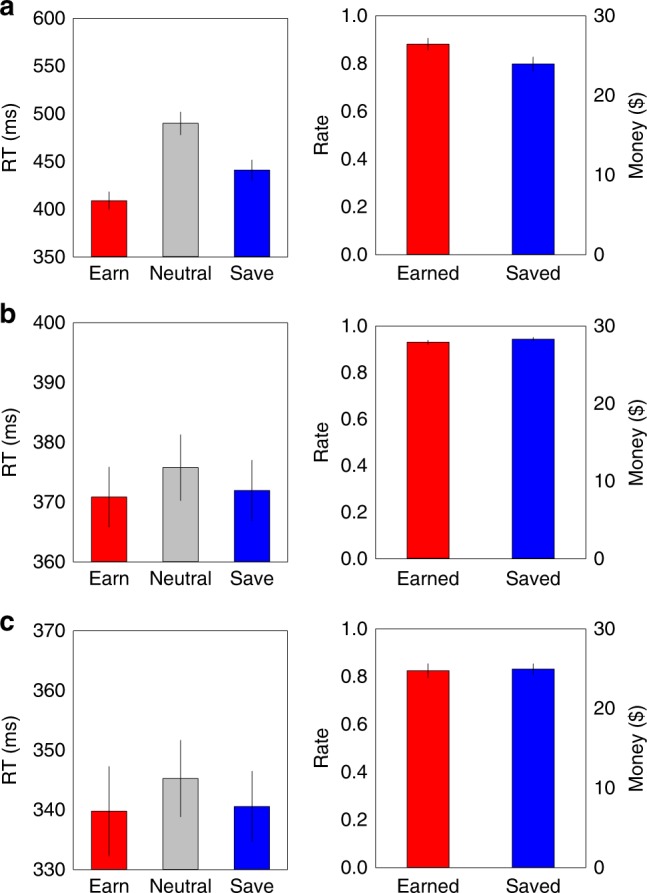
Value reinforcement task: response time, rate of correct responses, and payout. Top row (**a**): value reinforcement data in experiment 1a. Left panel, response time (RT, ms); right panel, successful earnings and savings in rate and cash. Middle row (**b**), value reinforcement data in experiment 1b; and bottom row (**c**), value reinforcement data in experiment 1c. For all, error bars indicate standard errors of the mean (SEM). See text for details

On value reinforcement, mean response time (RT) and error rate for color discriminations were separately submitted to a one-way repeated analysis of variance (ANOVA) with three levels (earn, save, and neutral). We used Greenhouse–Geisser correction when sphericity assumptions were not met in all ANOVAs. The RT ANOVA showed a significant main effect, *F*(1,21) = 20.67, *p* < 0.0001, *η*^2^ = 0.50, where colors associated with earning and saving elicited faster responses than neutral, which was taken as a more robust reinforced behavioral response. Earning also resulted in more robust speeded responses than saving: earn and neutral, *t*(15) = 6.01, *p* < 0.0001, *d* = 1.52; save and neutral, *t*(15) = 3.15, *p* = 0.007, *d* = 0.79; as well as earn and save, *t*(15) = 4.12, *p* = 0.001, *d* = 1.05, differed significantly (left panel, Fig. 2a). The ANOVA for error rate showed a significant main effect, *F*(2,30) = 6.79, *p* = 0.004, *η*^2^ = 0.31; revealing greater accuracy on earn relative to save trials, *t*(15) = 4.06, *p* = 0.001, *d* = 1.07. Further, taking into account the imposed RT threshold for payout, participants demonstrated high proportions of reinforcement, but higher for earning than saving (88% versus 80%, right panel, Fig. 2a), *t*(15) = 4.62, *p* < 0.001, *d* = 1.19.

**Fig. 3:**
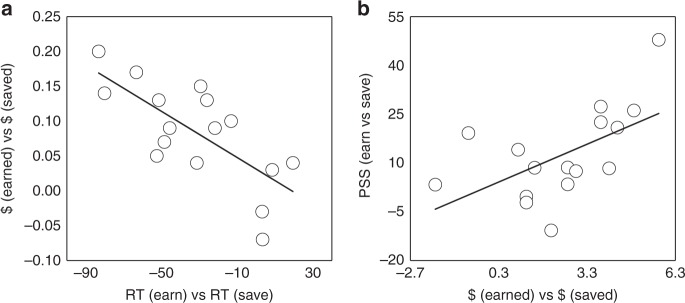
Differential behavioral and attentional salience for earning and saving. **a** Individual differences in behavioral responses (response time, RT) for earn versus save during reinforcement were related to the amount of money earned versus saved; **b** individual differences in reinforcement monetary advantage for earnings over savings were related to the magnitude of the earn-save asymmetry in temporal-order judgment, i.e., PSS (earn versus save) scores

Consistent with stronger reinforcement, the amount of money earned versus saved correlated with RT advantage for earn versus save, robust *R*^2^ = 0.482, *p* = 0.003 (Fig. 3a). Given the likelihood of success of reward (>80%), in combination with the RT and accuracy performance, both earnings and savings trials were rewarding and reflected reinforcement, relative to neutral. In addition, there was evidence of potential asymmetric reinforcement on earn compared to save conditions.

**Fig. 4:**
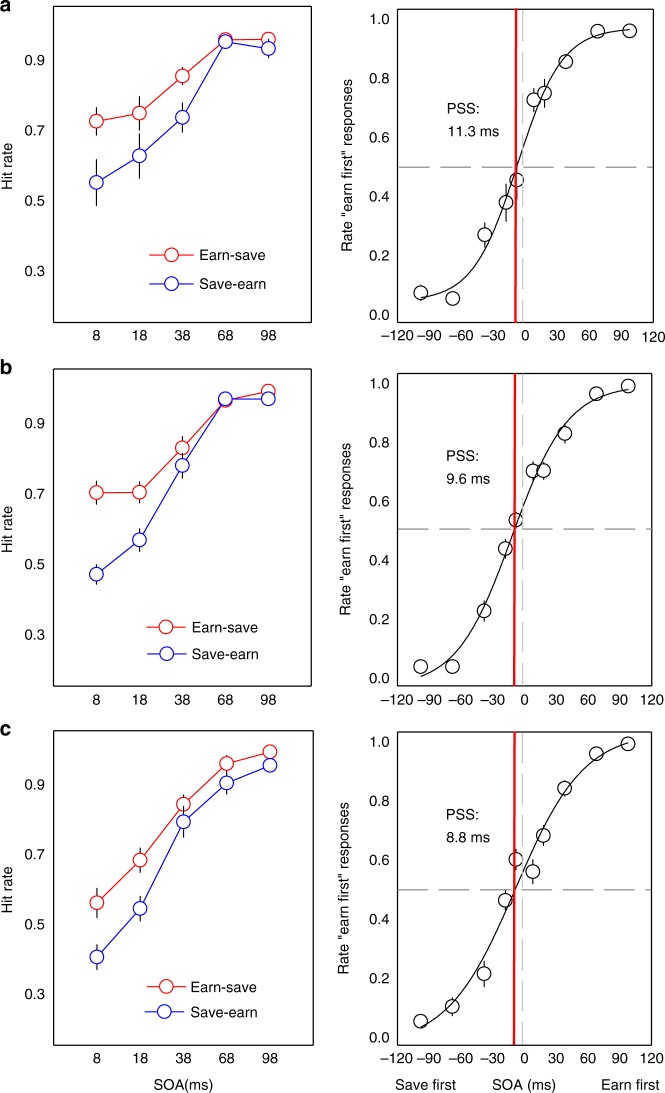
Temporal-order judgments for color cues predicting earning and saving. Top row (**a**): temporal-order judgment (TOJ) in experiment 1a. Left panel: the average hit rate for earn and save from the “earn-save” and “save-earn” trials as a function of stimulus-onset asynchrony (SOA). For the pairs in the trial, the first stimulus name appeared earlier physically; right panel: earn-save trials. Line is estimated point of subjective simultaneity (PSS) with leftward shift revealing save colors are judged to appear later. Middle row (**b**), TOJ in experiment 1b; and bottom row (**c**), TOJ in experiment 1c. For all, error bars indicate standard errors of the mean (SEM). See text for details

On TOJs, we first analyzed participants’ accuracy (for similar approach see ref.^52^) when earn and save were presented head to head, submitting hit rates to ANOVA: condition (earn first, save first) × stimulus-onset asymmetry (SOA; 8, 18, 38, 68, and 98 ms). The main effect for SOA, *F*(3,38) = 54.37, *p* < 0.001, *η*^2^ = 0.81, demonstrating decreased temporal-order accuracy with shorter-onset differences. A marginally significant effect of condition, *F*(1,15) = 4.31, *p* = 0.056, *η*^2^ = 0.22, revealed higher hit rates when earn colors were presented first relative to when save colors were presented first, specifically at shorter onsets (8 ~ 38), *F*(1,15) = 4.81, *p* = 0.044, *η*^2^ = 0.24, where onset information was limited (left panel, Fig. 4a).

To assess the effects of earning and saving on subjective appearance, we fit a Gaussian function to each individual’s data across onset asynchronies to estimate the PSS. Both earning and saving caused a shift in the psychometric function of TOJs, judged to appear prior to neutral colors without reinforcement^46,49^. Reinforcement inverted the judgment of temporal ordering: even when neutral color appeared first, participants judged reinforced colors to arrive earlier in time—a prior entry effect. Participants, on average, judged earn and save colors as arriving 16.6 and 15.6 ms prior to concurrently displayed neutral color, respectively: PSS_earn-neutral_, *t*(15) = 2.87, *p* = 0.012, *d* = 1.48; PSS_save-neutral_, *t*(15) = 2.92, *p* = 0.011, *d* = 1.51. However, when pitted against each other to compete directly for attention, colors associated with earning were judged to appear earlier in time than saving (PSS_earn-save_ = 11.3 ms), *t*(15) = 2.86, *p* = 0.012, *d* = 1.48 (right panel, Fig. 4a).

Prior studies have shown that altered attentional salience is directly related to preferential behavioral choices^17,18,43^. Looking across reinforcement and TOJ tasks, we also found that individual differences in final payout were related to TOJs, robust *R*^2^ = 0.286, *p* = 0.033 (Fig. 3b). The magnitude of the money earned versus saved advantage was associated with the earn-save asymmetry in TOJ. The decreased reinforcing power of save trials was associated with their delayed perception, potentially reflecting saving’s decreased attentional salience.

### Experiment 1b

We hypothesized that differential attention was central to savings posteriority. However, in experiment 1a, the reinforcement task required color discriminations and these same color discriminations were the basis for TOJs. As such, a reinforced “red” response during color discrimination may have increased the action value of that color^53,54^ influencing response selections in judgments of temporal order, rather than or in addition to reflecting increased attention and perceptual salience. To uncouple action and stimulus values, in experiment 1b participants now judged a left-right facing gap in the circles during reinforcement, rather than color. Gap side judgment had no correspondence with the color-reinforcement associations. As such, monetary reinforcement and color were rendered task and behavior irrelevant. Participants were now instructed to report on which side of fixation (left versus right) a stimulus arrived first, therefore temporal-order response selection was now entirely unrelated to reinforcement, i.e., color.

Performance in the reinforcement trials (gap side discrimination) revealed error rates were very low (mean < 1.4%). Earning and saving trials were faster than neutral, with a repeated-measures ANOVA demonstrating a significant condition main effect for RT, *F*(2,48) = 4.23, *p* = 0.025, *η*^2^ = 0.15; however, by removing the possibility for altered action values, there was now no difference between earning and saving, *t*(24) = 0.77, *p* = 0.45, *d* = 0.15 (left panel, Fig. 2b). This was also reflected in similarly high and matched proportions of earnings (93%) and savings (94%), *t*(24) = 1.89, *p* = 0.070, *d* = 0.38 (right panel, Fig. 2b). As such, with equivalent RTs, accuracy and absolute monetary reward, experiment 1b matched reinforcement in the earning and saving conditions.

Regarding TOJs, when earn and save were presented in head-to-head competition, hit rates were higher for earn (“earn-save”) first than for save (“save-earn”) first trials. The corresponding two-way ANOVA showed that a main effect of SOA, *F*(3,66) = 120.87, *p* < 0.001, *η*^2^ = 0.85; condition, *F*(1,22) = 13.25, *p* < .001, *η*^2^ = 0.38; and a condition × SOA interaction, *F*(3,65) = 11.33, *p* < 0.001, *η*^2^ = 0.34 (left panel, Fig. 4b). Once again, earning had greatest influence relative to saving at short SOAs (8 ~ 38), *F*(1,22) = 15.51, *p* = 0.001, *η*^2^ = 0.41, where there was diminished stimulus-driven temporal-onset information.

Estimating the PSS, prior entry was again observed for both earn and save colors, which were judged to arrive earlier than concurrently displayed neutral color: PSS_earn-neutral_, *t*(22) = 3.78, *p* = 0.001, *d* = 1.61; PSS_save-neutral_, *t*(22) = 3.13, *p* = 0.005, *d* = 1.34. Relative to neutral, PSS_earn-neutral_ was significantly larger than PSS_save-neutral_, *t*(22) = 3.13, *p* = 0.005, *d* = 0.79, and when earn and save colors competed directly, participants on average perceived earning as arriving 9.6 ms prior to concurrently displayed saving colors, *t*(22) = 4.10, *p* < 0.0001, *d* = 1.75 (right panel, Fig. 4b). Note that two participants were excluded from this analysis because of their PSS sizes were beyond three standard deviations from the mean. Despite changes from experiment 1a to the reinforcement and temporal-order tasks, and equating reinforcement, there remained a temporal posteriority for saving. That savings still lost out to earnings, after controlling for action salience, is consistent with differential acquired attentional salience for earning relative saving.

### Experiment 1c

Earning and saving appear to acquire differential attentional salience. This may reflect a failure to appreciate the equal magnitude of earnings and savings, defaulting to earnings having more salience. To ensure that earning and savings were equally salient magnitudes during reinforcement, we added a visual feedback display providing cumulative “gain” and “save” bars escalating in the same direction, and two different sounds were played for successful gains and savings, at the end of each value reinforcement trial (Supplementary Text and Supplementary Figure 1). Relatedly, examinations of positive and negative monetary reinforcement, as a model for earning and saving, may have different impact on subjective value. Earning and saving may reflect varying subjective utility for the individual, or even disutility. We now examine the subjective affective consequences of earning and saving and their relation to attentional salience. Additionally, we employed a stricter RT threshold for the reinforcement task used in experiment 1b, to increase risk^55^ and to ensure equivalent performance did not reflect a ceiling effect.

On value reinforcement, a repeated-measures ANOVA of RTs revealed a significant main effect of condition, *F*(2,36) = 3.29, *p* = 0.049, *η*^2^ = 0.16, with earn and save trials significantly faster than neutral (left panel, Fig. 2c), with no difference between earning and saving, *t*(18) = 0.63, *p* = 0.539, *d* = 0.16. An ANOVA of error rates was also significant, *F*(2,36) = 3.87, *p* = 0.030, *η*^2^ = 0.18, with the only statistically reliable difference existing between earning and neutral trials, *t*(18) = 2.58, *p* = 0.019, *d* = 0.62. When taking the stricter RT threshold into account, participants demonstrated an equal percentage of earnings (83%) and savings (83%), *t*(18) = 0.08, *p* = 0.935, *d* = 0.12, that was well below ceiling, consistent with equivalent reinforcement (right panel, Fig. 2c).

On TOJs, analysis of accuracy revealed a main effect of SOA, *F*(2,39) = 113.66, *p* < 0.001, and condition, *F*(1,18) = 8.62, *p* = 0.009, *η*^2^ = 0.32, demonstrating that when earn and save were presented in the same trial, the hit rates were higher for earn first than for save first trials. The two-way interaction was marginally significant, *F*(4,72) = 2.48, *p* = 0.051, *η*^2^ = 0.12, with earn versus save differences most pronounced at short SOAs (8 ~ 38), *F*(1,18) = 8.17, *p* = 0.010, *η*^2^ = 0.31 (left panel, Fig. 4c).

Prior entry was observed for earning and saving, as participants on average perceived earn and save stimuli as arriving 17.4 and 7.1 ms (PSS) prior to neutral stimuli, respectively: PSS_earn-neutral_, *t*(18) = 4.82, *p* < 0.001, *d* = 2.27; PSS_save-neutral_, *t*(18) = 2.42, *p* = 0.026, *d* = 1.14. Save colors demonstrated less temporal priority than earn colors, *t*(18) = 2.95, *p* = 0.009, *d* = 1.39. When earn and save colors were pitted directly against each other, participants on average perceived saving as arriving 8.4 ms following earning, *t*(18) = 2.89, *p* = 0.010, *d* = 1.36 (right panel, Fig. 4c).

Affect: Experiment 1c afforded examination of the subjective affective consequences of positive and negative monetary reinforcement. After the experiment, participants rated their subjective arousal and valence of the colored circles employed in the reinforcement task. A main effect of arousal, *F*(1,25) = 24.41, *p* < 0.0001, *η*^2^ = 0.49, revealed a common effect of earning and saving on increased arousal, *t*(18) = 5.51, *p* < 0.0001, *d* = 2.60, compared to neutral, consistent with their acquired salience. A main effect of valence, *F*(1,26) = 26.10, *p* < 0.0001, *η*^2^ = 0.50, revealed a distinct effects of earning and saving. Earning was associated with increased positive valence, *t*(18) = 3.43, *p* = 0.003, *d* = 1.62, while saving was associated with increased negative valence, *t*(18) = −5.62, *p* < 0.001, *d* = −2.65, relative to neutral. Thus, earning and saving had equivalent arousal and extremity of valence, but of opposing direction.

Summary analysis: Participants ascribe more value to things merely because they own them^14^. The salience of saving of one’s earnings in experiments 1a–c might be greatest when there are the most earnings to lose. While earnings are immediate, the salience of saving these earnings may accrue with time, i.e., with a larger bank account. The decreased attentional salience of saving may be, on average, diminished compared to earning because of this inequity. To have sufficient data to examine this, we conducted a summary analysis across studies, restricting analysis to the final quarter of experimental trials where there was an accumulated average potential payout of $19.50. With the most savings at stake, earnings (marginal gain of $.30) still outweighed savings (avoiding loss of $.30), with a highly reliable savings posteriority effect, PSS = 14.5 ms, *t*(57) = 6.33, *p* < 0.0001, *d* = 1.68.

### Experiment 2

In the experiments introduced above, we focused on positive and negative varieties of reinforcement as a model for earning and saving. Given the acquired negative valence and disutility of the saving condition, we would have expected greater attention^11,18,35^, yet we found a savings posteriority effect. Casting a condition as “savings” inverted what we would be expected from its equal arousal and increased negative value relative to earning. Thus, saving has connotations that counteract its negative value to be less salient.

Saving may be additionally influenced by a differing temporal perspective. Indeed, mirroring our evidence of a saving posteriority effect in perception, savings may be viewed as manifested later in the future, rather than the present^56^. Earnings and savings are likely understood to have different expected values now and in the future, reflecting a form of temporal discounting^27–29^. Accordingly, here we considered the earnings and savings as the immediate gratification of gains manifested in the moment versus gains that manifest at a future point in time. Applying the same paradigm as experiment 1c, we now cast savings and earnings in terms of positive reinforcement, both reflecting 10 cents gains. We manipulated the temporal perspective on earnings and savings. Using a simple framing manipulation, earning was presented as gains manifesting immediately (represented by a $0.10 as feedback), while saving was presented as gains manifested over trials, to come later (represented by an image of a piggy bank as feedback) (Supplementary Text and Supplementary Figure 2). Despite this intertemporal framing, earn and save trials accrued payouts shown at equal intervals (every 40 trials) and magnitudes. Savings were simply cast as earnings that were accrued and reserved for later use and therefore were currently inaccessible.

On value reinforcement, a repeated-measures ANOVA of RTs showed a nonsignificant effect, *F*(2,34) = 0.83, *η*^2^ = 0.05. Similarly, an ANOVA of error rate (mean = 3%) was not significant, *F*(2,34) = 0.78, *p* = 0.466, *η*^2^ = 0.04. Consistent with equivalent reinforcement, participants demonstrated an equal percentage of earnings (89%) and savings (90%), *t*(16) = 1.70, *p* = 0.11, *d* = 0.85. Note that one participant was excluded from this earning percentage analysis due to much lower percentage of earnings and savings than the rest of the participants (51% and 54%, respectively).

**Fig. 5:**
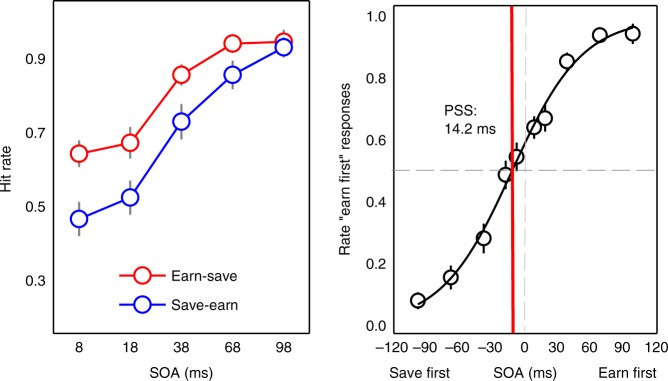
Temporal-order judgment where earning and saving were defined by temporal framing in Experiment 2. Left panel: the average hit rate for earn and save from the “earn-save” and “save-earn” trials as a function of stimulus-onset asynchrony (SOA). For the pairs in the trial, the first stimulus name appeared earlier physically. Right panel: earn-save trials. Line is estimated point of subjective simultaneity (PSS) with leftward shift revealing save colors are judged to appear later

On TOJs, analysis of accuracy revealed a main effect of SOA, *F*(3,44) = 86.35, *p* < 0.001, *η*^2^ = 0.86, and condition, *F*(1,17) = 6.37, *p* = 0.022, *η*^2^ = 0.27, demonstrating that when the earn and save stimuli were presented in the same trial, the hit rates were higher for earn first than for save first trials. The two-way interaction was not significant, *F*(4,68) = 1.78, *p* = 0.142, *η*^2^ = 0.10 (left panel, Fig. 5).

Estimating the PSS, prior entry was observed for both earning and saving, as participants, on average, perceived earn and save colors as arriving 24.3 and 13.4 ms (PSS) prior to neutral stimuli, respectively: PSS_earn-neutral_, *t*(17) = 3.92, *p* < 0.001, *d* = 1.90; PSS_save-neutral_, *t*(17) = 2.30, *p* = 0.026, *d* = 1.12. Earn colors again demonstrated more temporal priority than save colors, *t*(17) = 2.14, *p* = 0.047, *d* = 1.04. Consistent with the saving posteriority found in experiments 1a–c, when earn and save colors were pitted directly against each other, on average, participants perceived saving as arriving 14.2 ms following earning, *t*(17) = 2.31, *p* = 0.010, *d* = 1.12 (right panel, Fig. 5). Despite being matched in positive reinforcement, saving, i.e., earnings for later, was less attentionally salient and resulted in temporal posteriority, relative to immediate earnings.

After the experiment, participants rated their subjective arousal and valence of the colored circles employed in the reinforcement task. There was no significant effect of arousal between earnings, saving and neutral, *F*(1,21) = 1.98, *p* = 0.173, *η*^2^ = 0.09. A main effect of valence, *F*(1,20) = 6.83, *p* = 0.013, *η*^2^ = 0.26, revealed a distinct effect of earning and saving. Note that the Greenhouse–Geisser correction was employed here. Counter to experiment 1c, earning *t*(17) = 2.97, *p* = 0.009, *d* = 1.44; and saving *t*(17) = 2.34, *p* = 0.032, *d* = 1.14 were now both associated with increased positive valence relative to neutral, but did not differ significantly from each other, *t*(17) = 1.61, *p* = 0.126, *d* = 0.78. As such, now both save and earn were associated with positive valence, yet the temporal posteriority for savings persisted.

## Discussion

In the United States, despite strong motivation to earn, as reflected by a 96% employment rate, the savings rate is approximately 3%, with many having no savings^2^. While there are numerous complex factors that relate to the ability to save, in this study, we examined savings from a cognitive-affective perspective in a lab-based monetary reinforcement task. We examined how saving is subject to a more fundamental information-processing disadvantage as a target for moment-to-moment attention. According to two proposed models of earning and saving, we show how an orientation toward earning and saving influences one of the most basic judgments: when a stimulus appears. Colors predicting both earning and saving were judged to appear earlier in time than neutral outcome colors, whether color was task relevant (experiment 1a) or irrelevant (experiments 1b and 1c). However, when directly pitted against each other, colors predicting savings were consistently judged to appear later in time, a savings posteriority effect. The temporal priority for earnings over savings persisted when earning and savings were both modeled after positive reinforcement but framed as gains now or in the near future. These variants of reinforcement and the manipulation of temporal framing yielded a priority of earning over saving. This highlights that there are many potential mechanisms that can have a common influence on savings posteriority, reducing the moment-to-moment attentional salience of saving.

We show that colors, through association with earning and saving, acquire value and attentional salience relative to colors associated with neutral payouts. Operant conditioning classically is thought to strengthen an instrumental behavioral response, i.e., reinforcement^20^. It may also attentionally reinforce specific stimulus representations, resulting in their enhanced salience^57^. We show here one of the results of these reinforced stimulus properties is temporal priority, despite objective physical evidence to the contrary. We suggest that through greater attention, reinforcement biases elicit a response similar to increasing stimulus strength^47,58^, altering temporal decision processes to shape awareness.

Manipulation of task parameters across studies afforded a test of specific mechanistic accounts of savings posteriority. In experiment 1a, color was explicitly reinforced (i.e., money earned or saved based on fast and correct color discriminations) and was the feature reported in the TOJ (i.e., which color appeared first?). As such, temporal judgments may have reflected altered action and not attentional and temporal salience. By contrast, in experiments 1b and c, colors were implicitly associated with outcomes and not behaviorally reinforced—participants earned or saved money related to performance in judging the direction (left versus right) of a gap in colored circles. Color was also not the feature reported in the TOJ (which side, left or right, appeared first?), allowing a dissociation of action and attentional salience.

While earning and savings reinforcement differentially influenced action salience by altering the speed of behavioral responses (experiment 1a), reinforcement also specifically strengthened attention (experiments 1b, c, and 2) to bias temporal perceptions. Thus, relative to earning, saving was not only associated with decreased behavioral responsiveness, but also decreased attentional salience. It appears that, even when task irrelevant, we can acquire, compute, and compare utility at a stimulus-based level (for a similar opinion, see refs. ^59,60^). This asymmetric “gain approach” in attention to earnings, relative to  savings, is potent enough to subjectively distort objective temporal reality, and potentially one’s bank account.

We assert that rather than differential action values driving attentional engagement, our results are most consistent with the converse: attentional engagement and resulting salience can motivate differential behavioral selection^17,18,34,43^. Saving may have less salience than earning for attention and thus likely diminished inputs into choice^17,18^. This is consistent with evidence that attention can itself drive value and behavioral choice^34,61^. Individual differences in temporal salience were associated with the magnitude of payout in dollars, suggesting increased attentional salience can be translated to behavioral preference and money earned versus saved. Establishing the directionality between attention and choice, however, is difficult to determine by the present findings alone. Differential temporal salience was present even when we equated behavioral salience and payout for earning and saving (experiments 1b and 1c). As such, we suggest that attention is a more sensitive measure, preceding behavior change rather than resulting from it. In practice, relative inattention to saving would result in decreased choice to save, rather than the converse. This is consistent with evidence demonstrating how differential attention influences preference^41^, with attentional salience predicting consumer choice^42^.

Loss aversion refers to a preference for avoiding losses relative to acquiring equivalent gains, with a roughly 2.5-to-1 value^62^ for equal magnitude gains. According to prior work, loss aversion should align with our use of negative reinforcement, which should have greater subjective utility than positive reinforcement. However, we found colors associated with savings, modeled after negative reinforcement (avoiding loss of earnings), had acquired negative value. This disutility is somewhat surprising in the context of the greater utility of loss aversion. Unlike the direct experience evoked here, loss aversion is more pronounced in affective simulations or forecasting^13,63^. Across studies, we attempted to create equally potent positive and negative reinforcement manipulations, but consistently found that positive reinforcement received attentional priority.

While positive and negative reinforcement both engage reward circuitry^21^, in the present study, they had distinct affective consequences. Colors associated with gains acquired positive value, while those associated with avoiding losses acquired negative value. This may have reflected losses incurred in the savings condition, compared to gains missed in the earning condition, although both were the minority of trials (<20% gains missed and losses incurred). This negative valence should have resulted in more not less attention, according to negativity biases and outsized role of negative valence in cognition^35^. Despite this, savings still received less attentional priority, attesting to how the decreased salience of savings does not easily fit within existing models of how affect regulates attention^64^.

Evidence of decreased salience of savings is not easily understood as reflecting a more general principle arising from the two major affective factors that are known to modulate cognition and behavior: arousal and valence^64^. We show that depending on whether defined by negative reinforcement or positive reinforcement with temporal perspective, colors predicting savings acquire divergent affective responses—rendered more negative in valence (experiment 1c) or of equal positive valence (experiment 2)—yet, in all cases saving remained less attentionally salient than earning. The common acquired temporal salience of colors, associated with both earning and saving relative to neutral outcomes, does align with a well-characterized general role of arousal or valence extremity in attentional salience^65^. Yet, mean acquired arousal was equated across earning and saving colors, and reported arousal did not correlate with individual differences in earn-save asymmetry magnitude (*R*^2^ = 0.01). Biased in temporal salience of earnings over savings appear to represent a unique motivational contribution to these economic choices.

One special aspect of savings is its referencing of the future. Savings are typically reserved for future use, and thus are currently inaccessible. Temporal distance to future events modulates how concretely we can think about them^29^ as well as their valuation^27^, and imagination of the future has significant limitations^30^. Models of intertemporal choice reveal devaluation of future gains, particularly in the impulsive^66,67^. By contrast, cultures whose language grammatically connects the future with the present tend to foster future-oriented behavior, including saving more every year and retiring with more wealth^56^. When we modeled earnings and savings as gains manifesting immediately or later (i.e., after a number of trials), colors associated with earnings accumulating for later use, i.e., savings, remained less attentionally salient than immediate earnings. Just as savings are earnings are to come at a later time, the colors associated with savings were judged to appear later than colors associated with earnings. This savings posteriority effect provides evidence that temporal discounting also regulates attentional salience in the present, shaping perceptions and decisions. Importantly, in the present study, the delays were more psychological than real, and much shorter than the actual time to payout at the end of the experiment. As such, colors associated with earning and savings both acquired positive value of a similar magnitude. Even before evidence of temporal devaluation, the framing of gains as savings was sufficient to result in decreased attentional and temporal salience.

Whether resulting from variants of reinforcement or temporal perspective, earning and saving are subject to a basic asymmetry in attentional choice. While decreased attention to saving appears fundamental, present under a variety of conditions, it is nevertheless malleable. Indeed, we show simple colors acquire differential salience based on their reinforcement association and temporal reference frame. It has been shown that allowing people to interact with age-progressed renderings of themselves results in greater saving for the future^68^. Also, the ability to acquire a saving orientation is supported by cultural differences in savings^56^. One important limitation in the generalizability of the present studies is that they were performed in American undergraduate and graduate students. We predict that the savings posteriority effect presented here could be culture-dependent. If decreased saving arises from unequal attentional scales for earning and saving, then these scales can be rebalanced with appropriate attentional interventions, adding weight to saving one’s earnings in the present.

## Methods

### Participants

Across studies, 78 right-handed participants (30 males; mean age = 21) took part in the study and provided informed consent, as approved by the Institutional Review Board of Cornell University, Ithaca, NY. For additional information, please see Supplementary Methods.

### Procedures

In each experiment, participants started with value (monetary) reinforcement trials, where different colors were systematic associated with different monetary outcomes. Then, the experiments pseudo-randomly intermixed value reinforcement trials with TOJ trials of these same colors. For experiment 1a, participants performed a color discrimination task (red versus blue versus yellow) during the value reinforcement trials (Fig. 1a). Participants were informed that different colors represented an opportunity to gain or prevent the loss of monetary reward based on performance. The RT threshold for successful gain and loss avoidance was set at 650 ms based on pilot data. Participants made a TOJ on these same colors, indicating which color arrived first. TOJ tasks did not receive monetary reinforcement (Fig. 1b). The approach of experiment 1b was identical to experiment 1a, with two exceptions. First, during value reinforcement trials, participants now were instructed to perform a gap side discrimination task of the colored circles (left gap versus right gap in the circle). Second, during TOJ trials, now participants ignored color, but were instructed to report “on which side did the stimulus come first?”, left or right. This rendered color task irrelevant and removed the role of behavioral reinforcement on TOJ performance, to better isolate acquired attentional salience. Experiment 1c was identical to experiment 1b, with three exceptions. First, at the end of each value reinforcement trial, a visual feedback display provided cumulative “earn” and “save” bars. Second, we set a strict RT threshold (400 ms) for successful gain and loss avoidance, avoiding a ceiling effect for value reinforcement. Third, we examined subjective valence and arousal responses to colors following positive and negative monetary reinforcement.

Experiment 2 was similar to experiment 1c, with three main changes: first, in this experiment, both earn and save conditions allowed for gains, reflecting positive reinforcement; second, earnings remained immediate gains, with feedback signaled by $ 0.10 and “ka-ching” sound, while savings were explained as gains that they would receive in the near future, with the feedback display a piggy bank image and a coin clink sound, with accumulated payout bar display provided only three times for a run instead after each trial for both earnings and savings; and third, the monetary reward for each gain trial, whether it was an earn or save trial, was $0.10. For additional information, please see Supplementary Methods.

### Data analysis

For details, see the Supplementary Information. Performance in the value reinforcement task was measured by participants’ RTs on correct trials, error rates, and proportions of gains received and successful losses avoided. The significance level for the main statistical analyses was set at *p* < 0.05. Holm-Bonferroni correction was applied to the alpha criterion for multiple comparisons when determining significance. To assess the TOJ task, we calculated each participant’s PSS using a Gaussian fit method. We also analyzed participants’ accuracy (i.e., mean percent of correct responses) submitting hit rates to an ANOVA (temporal order of the presenting stimuli × SOA (8, 18, 38, 68, and 98 ms)^52^. Following Cohen^69^, effect sizes were reported as *η*^2^ (small, 0.01; medium, 0.06; large, 0.14) for ANOVAs, and as *d* (small = 0.20; medium = 0.50; large = 0.80) for planned comparison *t* tests.

### Data availability

The data that support the findings of this study are available from the corresponding authors upon request.

## Electronic supplementary material

Supplementary Information
