# Supplementary material for: Differential temporal salience of earning and saving
Source: Nat Commun. 2018 Jul 20;9:2843. doi: 10.1038/s41467-018-05201-9 (PMC6054624; doi:10.1038/s41467-018-05201-9)
Supplement: Supplementary file 1 — Supplementary Information [file 41467_2018_5201_MOESM1_ESM.pdf]

Differential temporal salience of earning and saving

Hu, Kesong [1,2], De Rosa, Eve [1,2], & Anderson , Adam K.[1,2]

1. Department of Human Development, Cornell University, Ithaca, 14853-4401, NY, USA
2. Human Neuroscience Institute, Cornell University, Ithaca, 14853-4401, NY, USA

Correspondence:

To whom correspondence should be addressed to Kesong Hu (hkesong@gmail.com) or Adam Anderson (anderson@cornell.edu).

Supporting Information

Supplementary Methods

Participants and Setting

Seventy eight right-handed participants took part in the study (Exp. 1a: N=16; Exp. 1b: N=25; Exp. 1c: N=19; Exp. 2, N=18; 30 males; mean age =21, range 18-33) and provided informed consent, as approved by the Institutional Review Board of Cornell University, Ithaca, NY. Subjects were free from psychiatric or neurological disease or related past history, as indicated via self-report. All reported normal or corrected-to-normal acuity and color vision, and all were naive to the purpose of the experiment. Participants were paid immediately after the experiment, both a nominal amount, that was topped up by their additional monetary incentive task winnings.

All experiments were conducted in a dimly lit room. The schedule of stimulus presentation and data collection were controlled by Presentation software (Neurobehavioral Systems, Albany, CA). Each participant was given a practice run of 30 trials that were not analyzed. Among this practice run, one half were discrimination task trials and the other half were TOJ task trials.

Experiment 1a

Value Reinforcement: Color Discrimination Task. During the reinforcement trials, participants performed a color discrimination task (red versus blue versus yellow). Each trial started with a fixation display for 1000 ms and was followed by a color circle (with left or right gap for Exps 1b, 1c, and 2) for 800 ms. The color red, blue and yellow were defined with RGB values as (255, 0, 0), (0, 0,255), and (255, 255, 0), which were all 5.5 lm/ft^2. An Extech Foot Candle light meter was

used to measure color luminance. There was a 1 – 5 second inter-stimulus-interval (ISI, mean: 2 sec) containing a blank screen that appeared after the target display. Participants were required to indicate if the circle was in red, blue, or yellow by pressing a corresponding button as fast as possible. Before the start of the experiment, participants were informed that with one of the color categories there would be an opportunity to gain or prevent the loss of monetary reward based on performance (100% contingency). On each *earn* trial, participants won $0.30 if they were both accurate and fast. They won $0.00 during error or slow trials. During *save* trials, participants avoided loss if they were both accurate and fast; otherwise they lost $0.30 for either slow or error trials. During neutral trials, participants did not gain or lose money regardless of their performance. A visual reward feedback (duration: 1 sec) with cumulative earnings was provided during the ISI display; concurrently, a sound was played acknowledging participants gain (beep sound 1) or loss (beep sound 2) of money. A different sound (sound 3) was played if participants provided an incorrect response. The RT threshold for a “fast” response was set at 650 ms based on pilot data. Note that earn, save, and neutral trials were balanced (33% each), and the earn and save colors were counterbalanced across participants..

Temporal Order Judgment. During the TOJ task, each trial started with an initial fixation display for 1000 ms. Subsequently, a colored circle (either in red, blue or yellow) was presented either on the left or on the right side of the fixation; this was, followed by a second circle of a different color, which appeared on the opposite side of the first circle after a pseudorandomly determined stimulus onset asynchrony (SOAs: 8, 18, 38, 68, and 98 ms). Participants were required to make a temporal order judgment, about which color circle was presented first, by pressing a corresponding key, indicating the color. Like value conditioning, the responses between red and blue were counterbalanced across participants. Unlike the discrimination task, speeded response was not emphasized for the TOJ task. Participants were informed that there

was no chance of gain or loss on TOJ trials. Finally, a jittered 1 – 5 second inter-trial interval (ITI, mean:2 sec) containing a blank screen terminated the trial. Note that the test stimuli used here were closed color circles, while during the reinforcement trials the stimuli had a gap on either left or right side.

The experiment included 5 runs, and each run consisted of 60 value reinforcement trials (20 gain, 20 loss, and 20 neutral) and 60 TOJ trials. Each run for the TOJ task utilized a 5 (SOA separating first and second stimulus: 8,18,38,68 and 98 ms) x 6 (value pairs: Earn-Neutral, Neutral-Earn, Save-Neutral, Neutral-Save, Earn-Save, Save- Earn; note the first mentioned of each pair appeared first) x 2 (left versus right side first) design. Trial order was pseudorandom.

Experiment 1b

The apparatus, stimuli, and approach were identical to Exp 1a, with two exceptions. First, during value reinforcement trials, participants were instructed to perform a gap side discrimination task (left versus right side gap). They were required to indicate "left" or "right" by pressing a corresponding left/right button as quickly and as accurately as possible. Unlike Exp 1a, the feature color-value association was orthogonal to the task, allowing us to separate encoding from response contributions. Second, during TOJ trials, participants were instructed to report "on which side did the stimulus come first?" by pressing a corresponding left/right key during the TOJ task. This removed any response correspondence between the discrimination and TOJ tasks, and response bias towards color in the TOJ task.

Experiment 1c

The apparatus and stimuli were identical to experiment 1b, with three additions. First, at the end of each value reinforcement trial, a visual feedback display provided cumulative "earn" and

“save” bars to increase their salience(*a progressing bar, Fig. S1*). Consistent with the visual feedback, two different sounds were played for successful earning and saving. Second, we set a strict RT threshold (400 ms) for successful gain and loss avoidance, avoiding a ceiling effect for value reinforcement, increasing the number of trials where subjects lost potential gains and did not avert potential losses. Third, we examined subjective valence and arousal responses following positive and negative reinforcement. Using 7 point scales, we measured each participant’s ratings of arousal (low to high) and valence (negative to positive) acquired for each of the presented color stimuli.

Experiment 2

The apparatus and stimuli were identical to experiment 1c, with several procedural changes. First, in this experiment, both earn and save meant gains. While savings were framed as future gains accumulating over trials, earnings were framed as immediate gains. Second, at the end of value reinforcement trials, “Earnings” received visual reward feedback of $0.10 (see left panel, Fig. S2) and cash register sound. For “Savings”, visual feedback was a piggy bank image (see right panel, Fig. S2) and the clink of a coin. The cumulative reward feedback (two separate progressing bars for earnings and savings, Fig. S1) for both “Earn” and “Save” trials was provided after every 40 trials. Third, we set a RT threshold (600 ms) for successful responses. On each earn and save trial, participants won $0.10 if they were both accurate and fast. They won $0.00 during error or slow trials. As such earning and saving only differed in their mental framing as manifestinging immediately or at a future time. During neutral trials, participants did not gain or lose money regardless of their performance. Finally, participants were instructed that they would be paid for what they earned immediately after the experiment, together with the base payment.

General Data Analysis

Our first hypothesis was that events associated with monetary reinforcement, both positive (earnings) and negative (savings), should bias judgment of temporal order. The second hypothesis, was when earnings and savings are pitted against each other, if earnings are more powerful than saving as a motivational factor, then events associated with earnings should receive temporal priority in perception over savings.

Performance in the value reinforcement task was measured by participants' reaction times (RTs), error rates, and proportions of gain receipt and successful loss avoidance. For each participant, mean RTs for correct response for each condition were calculated. RT and error rate data were submitted to a one-way repeated ANOVA with the factor "value reinforcement." In the present study, the Greenhouse–Geisser correction was used when the sphericity assumption was not met in ANOVAs[1]. Planned comparisons were then performed to test the value reinforcement effect between earn and save. Holm-Bonferroni correction was applied to the alpha criterion for multiple comparisons when determining significance.

To assess the TOJ task, we first analyzed participants' accuracy (for similar approach see Ref[2]) when earn and save were presented head-to-head, submitting hit rates to ANOVA: condition (Earn-first, Save-first) x stimulus onset asymmetry, SOA (8, 18, 38, 68 and 98 ms)]. Further, we calculated the participants' point of subjective simultaneity (PSS) by estimating the time interval between stimulus onsets needed by participants to perceive the two stimuli as arriving simultaneously[3–6]. In this way, we computed the mean proportion of trials in which the target was judged as "appearing first" in two conditions: 1) when it physically appeared first (denoted by SOA: 8, 18, 38, 68, and 98 ms); 2) when it physically appeared second (denoted by SOA: -8,- 18, -38, -68, and -98 ms). PSS was calculated using Gaussian fit method (the Gaussian function from Matlab, Mathworks, MA, USA).

For correlation analyses in the present study, we employed iterative reweighted least squares (the robustfit function from Matlab, Mathworks, Natick, MA, USA), given that standard Pearson correlation is sensitive to even a few influential data points[7,8]; results from robust regression were reported in terms of R^2. Significant correlations were further scrutinized via a randomization test in which the probability of each correlation was estimated non-parametrically by randomly shuffling the xy pairings (interactions =10,000). In general, significance level for main statistical analyses was set at $p < 0.05$.

Supplementary Figure 1

Illustration of the display sequence for value reinforcement task trials in Experiments 1b and 1c, and target stimuli examples (not drawn to scale). Each trial started with an initial fixation display, followed by a picture of a color circle image with a gap (here, a red circle with right gap). After the target stimulus, participants were informed about their potential reward or save for the trial, together with the accrued earnings and savings (lossess avoided) display. Between the target display and the feedback display, a variable-length inter-trial-interval was inserted (mean=2 sec).

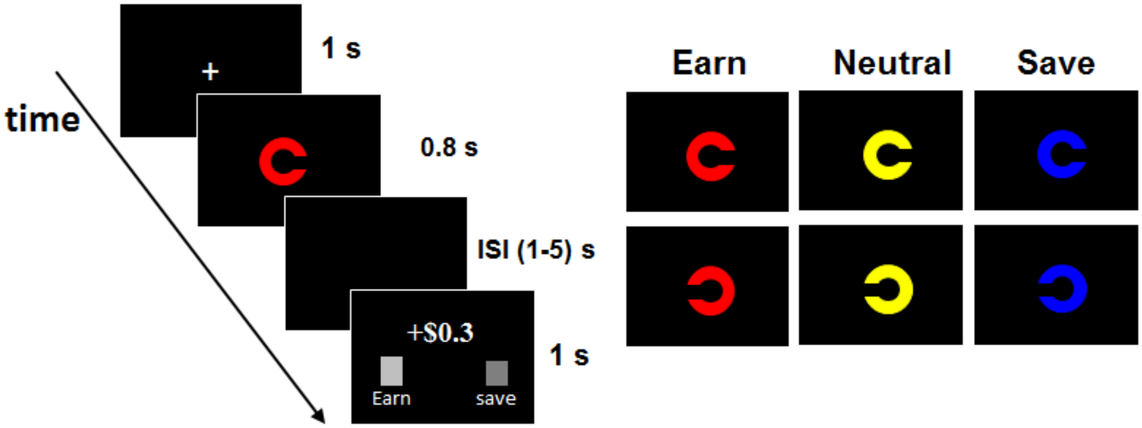

Supplementary Figure 2

Illustration of the display sequence for value reinforcement task trials in Experiment 2 (not drawn to scale). Each trial started with an initial fixation display, followed by a picture of a color circle image with a gap (here, a red circle with gap indicates an earn trial, while a blue circle with gap indictes a save trial, color counterbalanced across participants). After a left vs right gap response to the color circle stimulus, participants were informed about their potential to earn (panel a) or save (panel b) for the trial. The piggy bank image was created by Ken Teegardin and is reproduced here under a Creative Commons Attribution-Share Alike 2.0 Generic license. All rights reserved.

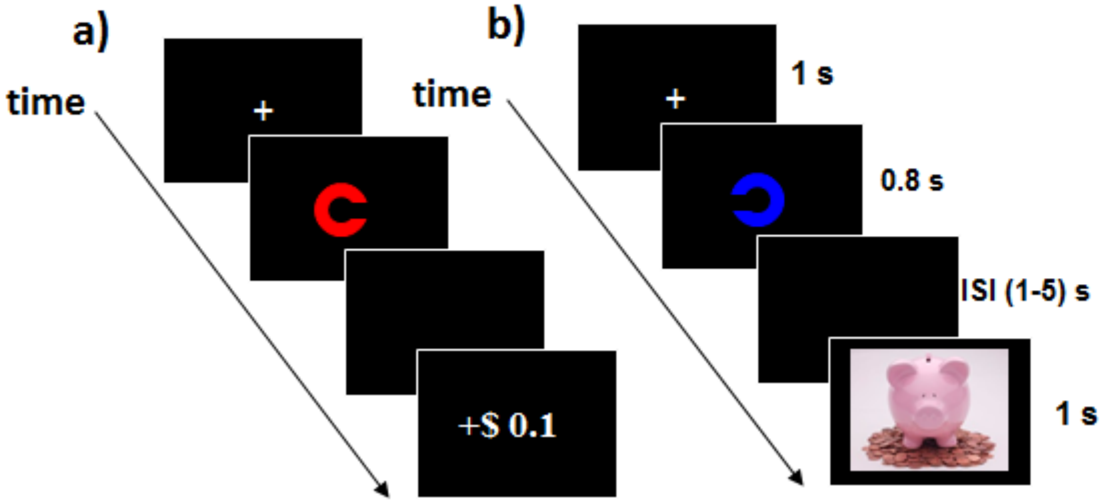

Reference

1. Jennings, J. R. & Wood, C. C. The e-adjustment procedure for repeated-measures analyses of variance. *Psychophysiology* 277–278 (1976).

2. Yarrow, K., Jahn, N., Durant, S. & Arnold, D. H. Shifts of criteria or neural timing? The assumptions underlying timing perception studies. *Conscious. Cogn.* **20,** 1518–1531 (2011).

3. Shore, D. I., Spence, C. & Klein, R. M. Visual prior entry. *Psychol. Sci.* **12,** 205–212 (2001).

4. Theeuwes, J. & Burg, E. Van Der. Priming makes a stimulus more salient. *J. Vis.* **13,** 1–11 (2013).

5. West, G. L., Anderson, A. K., Bedwell, J. S. & Pratt, J. Red diffuse light suppresses the accelerated perception of fear. *Psychol. Sci.* **21,** 992–999 (2010).

6. West, G., Anderson, A. & Pratt, J. Motivationally Significant Stimuli Show Visual Prior Entry : Evidence for Attentional Capture. *J. Exp. Psychol. Hum. Percept. Perform.* **35,** 1032–1042 (2009).

7. Wager, T. D., Keller, M. C., Lacey, S. C. & Jonides, J. Increased sensitivity in neuroimaging analyses using robust regression. *Neuroimage* **26,** 99–113 (2005).

8. Wilcox, R. R. Outlier detection. *Encycl. Stat. Behav. Sci.* (2005).
